# Corticospinal excitability as a biomarker of myofascial pain syndrome

**DOI:** 10.1097/PR9.0000000000000594

**Published:** 2017-04-18

**Authors:** Aurore Thibaut, Dian Zeng, Wolnei Caumo, Jianhua Liu, Felipe Fregni

**Affiliations:** a Spaulding Neuromodulation Center, Spaulding Rehabilitation Hospital, Harvard Medical School, Boston, MA, USA; b Guangdong Hospital of Traditional Chinese Medicine, Guangzhou University of Chinese Medicine, Guangzhou, China; c Laboratory of Pain and Neuromodulation, Hospital de Clinicas de Porto Alegre, Porto Alegre, Brazil

**Keywords:** Myofascial pain syndrome, Disinhibition, Intracortical inhibition, Intracortical facilitation, Cortical excitability, Pain, Motor cortex

## Abstract

**Introduction:**

Myofascial pain syndrome (MPS) is a common chronic pain disorder that lacks effective diagnostic criteria. To better understand neurophysiological changes in chronic pain, several trials exploring corticospinal excitability in different populations of patients with chronic pain have been performed.

**Objectives:**

In this systematic review, we aimed to investigate the current literature on MPS and intracortical disinhibition, by means of increased intracortical facilitation and decreased intracortical inhibition (ICI).

**Methods:**

We performed a search on PubMed to identify clinical trials on MPS and transcranial magnetic stimulation measurements. We then applied the Harford Hill criteria to the identified studies to assess the possible causal relationship between intracortical disinhibition measurements and MPS. Finally, we compared our findings on MPS with other chronic pain conditions.

**Results:**

Four studies assessing corticospinal excitability in patients with MPS were found. Although the amount of trials available is limited, all the reported studies indicated an increased intracortical disinhibition in patients with MPS. Importantly, these measurements were also correlated with psychological factors, such as pain catastrophism, or anxiety. However, based on the Harford Hill criteria, we could not assert a strong causal relationship between these markers and MPS. Although intracortical disinhibition has been consistently found in patients having MPS, this lack of cortical inhibition was not only observed in this specific chronic pain syndrome but also in fibromyalgia and neuropathic pain conditions.

**Conclusion:**

Intracortical disinhibition seems to be a marker that has been consistently observed in MPS. Future prospective cohort studies could provide new insights in the development of neoplastic and maladaptive changes occurring in chronic pain syndromes and help the development of new therapeutic options.

## Abstract 2

Intracortical disinhibition, by means of decreased intracortical inhibition and increased intracortical facilitation, seems to be a reliable marker of chronic myofascial pain syndrome.

## 1. Introduction

Myofascial pain syndromes (MPSs) are a group of painful conditions that affect muscles and connective tissues.^26^ These musculoskeletal disorders involve the sensory, motor, and autonomic systems and are characterized by skeletal muscle fibers nodules, which are called myofascial trigger points (MTrPs).^22^ These MTrPs are characterized by focal muscle hyperirritability to a sustained stimulus, and involve, among others, the integration of cellular signaling, neuromuscular inputs, excitation-contraction coupling, and the hemodynamic system.^26^ These sustained nociceptive stimuli induce the apoptosis of inhibitory neurons at the segmental levels affected by the peripheral noxious input, and such an action can sensitize dorsal horn neurons leading to allodynia, hyperalgesia, temporal summation of pain, and expanded pain patterns.^4^ Myofascial trigger points represent a major clinical sign of MPS that differentiate this syndrome from other chronic pain syndromes such as fibromyalgia and neuropathic pain syndromes.^20,21^ Traumatic events, muscular overloads, psychological stress, and systemic pathology may lead to the development of MTrPs. These MTrPs can either resolve spontaneously or become chronic, at which point, patients may develop MPS. However, the exact pathophysiology of MPS development is still not entirely understood. As with other chronic pain syndromes, it is particularly difficult to determine the underlying mechanisms of MPS given that pain involves the nervous system at various levels: cortical areas, the limbic system, the thalamus, the spinal–bulbospinal loop, and the spinal reticular tract; all constitute the so-called “pain matrix”.^64^

In chronic pain syndrome, cortical alterations are frequently found, with the motor cortex as the most commonly reported cortical area. Corticospinal excitability, measured by transcranial magnetic stimulation (TMS), represents a valuable tool to better characterize neurophysiological mechanisms of chronic pain.^34^ A valuable biomarker in chronic pain is intracortical disinhibition, as measured by intracortical inhibition and intracortical facilitation (ICI and ICF, respectively; for a review Ref. 23). A decrease in ICI could indicate lack of inhibition, or disinhibition, and an increase in ICF. This disinhibition is thought to be a determining factor in chronic pain syndromes,^23^ leading to a hyperexcitability of the motor cortex. Modifications of these cortical excitability parameters may also be influenced by the chronicity of the condition, the severity of symptoms, medication, and additional factors such as psychiatric disorders. Other neurophysiological methods, such as functional magnetic resonance imaging (fMRI) and event-related potential, also demonstrated these cortical changes in chronic pain (for recent reviews Ref. 42, 49). For instance, patients with fibromyalgia present a different activation pattern as compared to healthy subjects during both nonnociceptive sensory and painful stimulations and show an excess of cortical activity.^5,12,50^

As intracortical disinhibition is a reliable marker of motor cortical excitability dysfunction as indexed by TMS (specifically ICI and ICF alteration), the purpose of this systematic review was to first analyze these findings, and second, to explore and discuss their meanings in the context of mechanisms of pain in MPS. Therefore, we (1) defined intracortical disinhibition as indexed by TMS and its relationship with pain; (2) reviewed the findings of clinical trials assessing corticospinal excitability in MPS; (3) evaluated the causal relationship between findings of intracortical disinhibition and MPS based on Bradford Hill causality criteria; and (4) discussed if these measurements of intracortical disinhibition are specific for MPS.

## 2. Primary motor cortex disinhibition as indexed by transcranial magnetic stimulation and its relationship with pain

### 2.1. Parameters of motor cortex excitability: understanding their meaning

Motor-evoked potential (MEP) is a muscular response recorded using electromyography, when an electrical or magnetic stimulation is evoked, for instance using TMS, over the motor cortex. This measurement is used to describe the motor nerve excitability and integrity and reflects the global corticospinal excitability. Resting motor threshold (MT), which is another measure of global corticospinal excitability, is defined as the minimum TMS intensity sufficient to produce a predefined MEP in the contralateral targeted muscle in at least 50% of trials with an amplitude of 50 mV.^52^

The cortical silent period (CSP) refers to the refractory period occurring after the action potential. To record it, a suprathreshold stimulation is applied over the contralateral motor cortex during voluntary contraction of a target muscle, resulting in a period of electromyographic silence for up to several hundred milliseconds.^25^ A decrease in CSP latency can be interpreted as lack of inhibition or disinhibition. Pharmacological interventions suggest that CSP is, in part, related to the activity of the gamma-aminobutyric acid (GABA)ergic neurotransmitter system. Several trials reported a prolongation of the CSP latency when subjects were given baclofen (GABA_B_ agonist).^59,61^ Short interval intracortical inhibition (SICI) is another parameter of cortical excitability. It is measured by paired-pulse TMS, during which a pulse is delivered after 1 to 6 milliseconds after a subthreshold stimulation.^72^ The amplitude of the MEP of the second pulse is usually found to be reduced.

The physiological mechanism for SICI is thought to be a GABA_A_ receptor–mediated inhibition of motor cortex output cells.^15,71^ Indeed, it has been shown that its duration is enhanced by benzodiazepines, which are allosteric positive modulators of the GABA_A_ receptor.^15,71^ The duration of SICI is approximately 20 milliseconds, which is similar to inhibitory postsynaptic potential.^2^ Because SICI is modulated by drugs that enhance the GABA_A_ inhibitory system,^27,70^ the SICI duration is thought to be linked to inhibitory postsynaptic potential from the GABA_A_ receptor stimulation.^37^

Regarding intracortical ICF, similar paired-pulse TMS is used, with a pulse delivered 6 to 20 milliseconds after a subthreshold stimulation.^72^ In this case, an increase in the pulse amplitude is usually found, as compared to a single test pulse. Intracortical facilitation originates from excitatory postsynaptic potentials mainly mediated by glutaminergic *N*-methyl-d-aspartate (NMDA) receptors.^43^ However, the physiological and mechanisms for ICF are still not entirely understood. It has also been hypothesized that ICF is not only mediated by excitatory mechanisms but by a balance between inhibition and excitation.^51^

### 2.2. Relationship between M1 and pain

Pain is a complex phenomenon involving multiple nervous system functions, including sensory, affective, cognitive, and motor components. The specific interactions between pain and the motor cortex are not yet fully understood. However, that brain regions involved in motor function, such as the primary motor cortex, supplementary motor area, the anterior cingulate cortex, the lenticular and caudate nuclei, and the cerebellum, have expressed metabolic modifications after a painful stimulus.^48^

Although the primary motor cortex (M1) is not part of the pain matrix, it is considered to play a key role in modulation of pain in different chronic pain syndromes. M1 has wide connections to some of the sensory relay nuclei in the thalamus and to efferent and afferent fibers in the spinal cord responsible for transmission of painful stimuli and modulation of motor response to noxious stimuli. Many trials on patients with chronic pain have identified a maladaptive plasticity process in this specific cortical area.^39,54^ In addition, studies on both animal models and humans have shown that modulation of M1 activity induces important analgesic effects. For instance, simply observing a moving hand has been shown to increase the pain threshold, whereas observing a still hand decreases the threshold.^67^ This allows us to hypothesize that M1 can act as a modulator of pain processing, and therefore, influencing its baseline activity could lead to changes in pain perception in chronic pain syndromes. Different types of interventions such as noninvasive brain stimulation (NIBS) techniques can target this cortical area, to modulate its activity in order to modify its output responses regarding pain perception (for a review Ref. 32).

### 2.3. Relationship between intracortical disinhibition and pain

The most common hypothesis of chronic pain syndromes mechanisms, such as fibromyalgia, is based on the central sensitization theory and is related to a dysfunction in the extensive neural circuit responsible for processing pain. One such hypothesis, central sensitization, is a proposed physiological phenomenon in which central nervous system neurons become hyperexcitable, or less inhibited, because of modifications of peripheral and central nervous system as a consequence of chronic noxious stimuli,^44^ resulting in hypersensitivity of both noxious and nonnoxious stimuli.^68^

The phenomenon of disinhibition, by means of increase in ICF and/or decrease in ICI, is thought to be the consequence of a dysregulation of GABA-dependent inhibition and NMDA-dependent excitatory circuits.^70,72^ These changes in cortical plasticity could be explained as a compensatory mechanism to downregulate increased excitability in the pain neural networks such as thalamic structure, as previously hypothesized.^6^ Although cortical disinhibition can be interpreted as a decreased ICI and/or an increased ICF, it should be noted that various differences exist between these 2 parameters in terms of neurobiological bases, neurotransmitters involved, or thresholds to obtain. These should be taken into account when analyzing and interpreting the results of a trial.

When the intensity of a painful stimulus increases, the pain-inhibitory system can be disrupted as a summation effect. This cortical disinhibition phenomenon can amplify the intensity of pain signals from the peripheral nervous system to the neural pain matrix and, thus, can lead to increased pain sensation.^3^ In fact, lack of motor activity can enhance this mechanism by a feedback loop. This finding has been observed in MPS, and in conditions such as fibromyalgia and migraine, whereas it has not been identified in nociceptive pain such as osteoarthritis.^8^ This gives us a better understanding on how to develop treatments for neuropathic chronic pain, by increasing ICI or decreasing ICF. For instance, NIBS technique, particularly repetitive TMS (rTMS), has been shown to reduce pain and disinhibition in patients with neuropathic pain,^33^ whereas for patients having nonneuropathic pain who do not express cortical disinhibition, rTMS did not induce the same analgesic effect.^57^

## 3. Human studies evaluating corticospinal excitability in myofascial pain syndrome

To analyze intracortical disinhibition in MPS, we performed a search on PubMed using the following terms: “myofascial pain syndrome” and “TMS” or “Transcranial Magnetic Stimulation” or “cortical excitability”. We also looked at the reference list of the articles we retrieved to check if there were any additional articles. We also contacted experts in the field of MPS and TMS. Finally, we looked at neuromodulation meetings proceeding to check if there was any unpublished manuscript on MPS and cortical excitability.

We included articles involving patients with MPS and assessing cortical excitability. We excluded articles that were not focusing on MPS or articles that did not report cortical excitability measurements. Reviews and articles not written in English were also excluded.

**Figure 1.**
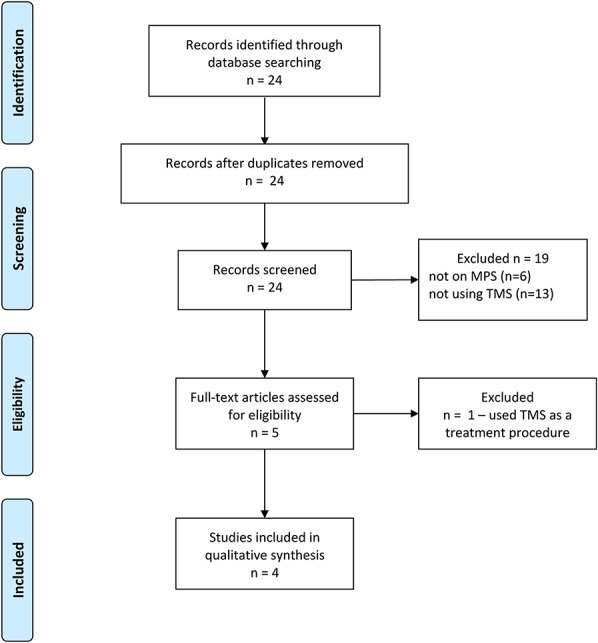
PRISMA flow chart describing identification and selection of studies for this systematic review.

Of the 24 articles researched, 4 were found to investigate cortical excitability in MPS (Fig. 1).

### 3.1. Cortical excitability of the motor cortex in myofascial pain syndrome and its relationship with pain catastrophizing

In a study conducted by Volz et al (2013), we aimed to explore the relationship between a neurophysiological marker of cortical excitability, as assessed by TMS, and catastrophizing, as assessed by the Brazilian Portuguese Pain Catastrophizing Scale (B-PCS), in patients with chronic MPS. We included 24 patients having MPS (all women, age 48 ± 13 years).^66^ In this study, MT, ICI, and ICF measurements were collected to assess cortical excitability and compared the results with patients' level of pain catastrophizing and pain as measured by pain pressure threshold. Intracortical facilitation was found to be significantly associated with pain catastrophizing, whereas SICI was associated with pain pressure threshold scores, with positive correlation between the 2 factors (decrease in SICI was related to lower pain threshold). In addition, ICF was positively correlated to the magnitude of pain (severity, interference with daily activity, and emotional burden, as measured by the Profile of Chronic Pain: Screen for a Brazilian Population–B-PCS^9,53^). In conclusion, we suggested that glutamatergic activity may be associated with mechanisms underlying pain catastrophizing. These results are in line with previous findings on other chronic pain pathologies, such as fibromyalgia, reporting impaired ICI.^34,36^

### 3.2. Relationship between anxiety and intracortical inhibition and pain modulation in myofascial pain syndrome

In the second study by Vidor et al (2014), we aimed to investigate how anxiety could influence cortical excitability, as quantified by CSP, ICI, and ICF, and pain modulation, as measured by conditioned pain modulation (CPM).^65^ Forty-seven patients with MPS (47 women, age: 47 ± 11.5 years) were included in this study. We found that anxiety was positively correlated with ICF and negatively correlated with CSP. In addition, we observed that the interaction between pain and anxiety reduced the CPM responses. This suggest that the function of the descending modulatory system was reduced when there was increase disinhibition, as already shown in other chronic pain conditions.^31,55^ When assessing the correlation between B-PCP and cortical excitably markers, a positive correlation with ICF was found, whereas no correlations with ICI nor CSP were identified. This study showed that the influence of anxiety on pain is linked to central sensitization of nociceptive neurons, which contribute to an aggravation of chronic pain symptoms.

### 3.3. Relationship between cortical excitability, descending pain modulation system, and brain-derived neurotrophic factor in myofascial pain syndrome

Our third article, from Botelho et al (2016), investigates the relationship between neurophysiological, neurochemical, and clinical outcomes in MPS. We studied the relationship between descending pain modulation (ie, CPM), corticospinal excitability, and brain-derived neurotrophic factor (BDNF) as a marker of neuroplasticity, in MPS.^3^ In this cross-sectional study, we investigated the relationship between cortical excitability and BDNF levels in 33 women with chronic MPS who responded (n = 10, age: 48 ± 9 years) and did not respond (n = 23, age: 43 ± 15 years) to CPM. Nonresponders had an increase in ICF and higher BDNF levels as compared to responders. In addition, nonresponders expressed greater disability related to pain and a lower level of heat pain threshold. As in the second study, no effect was observed on ICI between responders and nonresponders, nor between pain assessments (B-PCP) and this cortical parameter. The authors suggested that the increased central sensitization related to chronic pain leads to a loss of descending pain inhibition, or disinhibition, that would cause compensatory mechanisms shown by an increase in cortical excitability. They also proposed that BDNF level could be a marker of central sanitization, which is caused by enhanced membrane and synaptic activity, combined with a decrease in inhibition in the somatosensory system.^30^

### 3.4. Comparison of cortical disinhibition patterns in myofascial pain syndrome and other chronic pain syndromes

In this fourth study, by Caumo et al (2016), we aimed to better understand the mechanisms associated with the central sensitization, as it is a critical component of chronic pain syndromes. In this cross-sectional study, we included 114 women, aged 19–65 years old, with chronic pain syndromes. Nineteen were diagnosed with fibromyalgia, 54 with MPS, and 27 with osteoarthritis, as well as 14 healthy subjects. We assessed serum BDNF level, motor cortex excitability parameters, and change on pain sensation during CPM task.^8^ Patients with MPS presented higher SICF and lower SICI and CSP, as compared to healthy controls. Except for MEP, which is more elevated in MPS subjects, the other parameters of intracortical disinhibition were similar to those observed in patients with fibromyalgia. Patients with osteoarthritis expressed different cortical excitability patterns when compared with the other 2 conditions, results closer to what was observed in healthy controls. We also identified that BDNF levels were negatively correlated with SICI and the level of pain during CPM. These findings suggest that patients with MPS and fibromyalgia have a larger disinhibition pattern in the motor cortex and the descending pain-inhibitory system than that of patients with osteoarthritis. This means that the disinhibition of the motor cortex and the dysfunction in the descending pain regulatory system is more severe in patients without tissue injury (MPS and fibromyalgia) than in patients with structural lesions (osteoarthritis). Based on these results, we proposed that increased BDNF levels might also be involved in these pathological processes (disinhibition of motor cortex and dysfunction of descending inhibitory pain modulation system), but independently from the physiopathological mechanisms of musculoskeletal chronic pain syndromes.

The results of these 4 studies confirmed that disinhibition of the motor cortex, by means of an increase in ICF or a decrease in ICI, is a central marker of MPS. These markers were reproducibly altered in these 4 trials, which make them reliable biomarkers of this pathology. In addition, factors such as anxiety or pain catastrophizing and enhanced pain symptoms were correlated to a greater dysregulation of cortical inhibition.^65,66^ Increased cortical disinhibition was also associated with increased BDNF levels, which could be linked to synaptic plasticity, inducing a higher nociceptive transmission.^3^ Finally, cortical inhibition and facilitation in different populations of patients with chronic pain, both without tissue injury (MPS and fibromyalgia), and with structural lesions (osteoarthritis), different patterns were identified.^8^ Patients with structural lesions did not present this lack of cortical inhibition, whereas patients with fibromyalgia demonstrated the same dysregulation than patients with MPS, except for MEP, which represents the excitability of corticospinal tracts.^47^ It has been hypothesized that MPS and fibromyalgia are part of the same syndrome but at different time points, explaining the highly similar cortical excitability pattern between these 2 conditions.^17^ It should be noted that all 4 studies included female participants only. Therefore, we cannot generalize these findings to the entire population of patients with MPS, although women tend to be more often affected than men.^19^ The meaning of these biomarkers of intracortical disinhibition, and the similarities and differences between several chronic pain syndromes, will be further discussed in the next sections. In addition, it should be emphasized that all these studies were performed by our group, and therefore there is a lack of parallel work from other research investigators that could confirm our findings.

## 4. Is there a causal relationship between measurements of intracortical disinhibition and myofascial pain syndrome?

Here we aimed to evaluate the causal relationship between ICI and MPS using a qualitative approach to better characterize the validity of ICI as a biomarker for MPS. However, it needs to be acknowledged that it is possible that a causal relationship between these 2 variables does not exist, although ICI may still be useful as a marker of treatment, for example. To determine whether a causal relationship between a decrease in ICI and level of pain in MPS exists, we applied the Bradford Hill criteria^24^ to our findings. Bradford Hill defined 9 criteria (strength, consistency, specificity, temporally, biological gradient, plausibility, coherence, experiment, and analogy). As only 4 trials assessed this relationship (see previous section), we could not translate their findings to all the criteria listed above.

### 4.1. Strength

Although the effect size of the correlations was not reported in all studies, we can evaluate the strength with the *R*^2^ of the models assessing the correlation between ICI or ICF and pain measurements or the Pearson correlation coefficient. In the first study, the *R*^2^ for the ICI and pressure pain threshold model was 0.19 (pain threshold explains 19% of the variance of the ICI).^66^ Only a trend to significance was found for ICF. The second study showed an effect size of 0.19 in a model assessing the correlation between ICF and pain measurements.^65^ A similar relationship between ICF and pain measurements (Pearson correlation coefficient *r* = 0.45; *P* < 0.01) was identified in the third trial, whereas no significant correlation was identified for ICI nor CSP.^3^ For the last study, the authors demonstrated that patients with MPS express a significant decrease in ICI and CSP and increase in ICF, as compared to healthy controls.^8^ These results demonstrate that intracortical markers of disinhibition represent an important factor of pain control and modulation in patients with MPS; however, to date, these studies show a mild strength of this association.

### 4.2. Consistency

Two studies reported an association between pain measurements and ICF, one between pain and ICI, and the fourth one demonstrated that patients with MPS express decreased ICI and increased ICF as compared to healthy controls. If we consider ICI and ICF as markers of cortical disinhibition, all studies demonstrated the association between either pain and intracortical disinhibition or the lack of inhibition in patients with MPS, compared to healthy controls. These reported results are consistent and reproducible through the 4 trials investigating intracortical disinhibition in patients with MPS.

### 4.3. Specificity

As observed in Caumo et al (2016),^8^ patients with fibromyalgia and MPS share some similarities because both pathologies expressed a reduction of ICI and an increase in ICF. In other etiologies, such as chronic pain after limb amputation,^10,58^ patients with complex regional pain syndrome,^35,56^ or neuropathic pain syndromes,^57^ there has also been reports of cortical disinhibition. Therefore, the specificity can be considered low.

### 4.4. Plausibility

The disinhibition phenomenon occurring in MPS is in line with the sensitization theory. Central sensitization involves a proliferation of synaptic activity due to trophic factors to support maladaptive plasticity that perpetuates the sensation of pain. Intracortical disinhibition is the consequence of an imbalance between the excitatory and inhibitory system, mediated by the reduction and activation of GABA and NMDA, respectively.^46^ Consequently, this lack of inhibition in ICI in patients with MPS explains the observed increase in ICF and decrease in ICI, and with regards to the primary motor cortex, is also understood to function as an important pain modulator. Therefore, plausibility can be considered high.

### 4.5. Coherence

When looking at animal studies, only a few trials used a rat model of MPS. One study investigated the role of GABA_A_ receptor expression in MPS and demonstrated the reduction of these receptors in rats with MPS,^29^ which is in line with the theory of disinhibition in humans, as evaluated by intracortical excitability measurements.

### 4.6. Experiment

One TMS trial investigated the effect of excitatory rTMS on pain relief in patients with MPS.^14^ In this study, the authors showed that 10 sessions of rTMS decreased pain and induced a reduction of ICF by 24%. However, no effect on SICI and CSP could be detected. This experiment demonstrated that a reduction of pain could be linked to a normalization of intracortical measurement and a reduction of intracortical disinhibition. This relationship has been found in other neuropathic pain syndromes. For instance, Lefaucheur et al (2006) identified that a decrease in ICI was correlated with pain relief, suggesting a restoration of defective intracortical inhibitory processes.^33,34^ Thus, measurements of intracortical disinhibition seem to be a reliable marker of chronic pain for some specific etiologies (ie, absence of structural pathology).

### 4.7. Analogy

Other markers of neuronal activity such as electroencephalogram or fMRI have been used to better understand the mechanisms of various pain conditions. However, so far only one study used fMRI to assess functional connectivity, white matter integrity using diffusion tensor imaging, and fractional anisotropy in patients with MPS.^40^ The authors found hypoconnectivity in the frontoparietal attention network in patients with MPS as compared to healthy controls. These findings demonstrate the abnormal cortical activity in patients with MPS; however, they are not related to an increase in cortical excitability or activity and do not emphasize the mechanisms of cortical disinhibition in this population of patients.

As mentioned by Fedak et al, 2015, the causal relationship between a condition and a factor can be very complex because diseases are the results of the interaction and balance between multiple factors.^16^ In our analysis, we did find some factors that would support a causal role, such as coherence, plausibility, and experiment. However, there are only a few studies to test for these factors and criteria, such as strength, specificity, and consistency, which are also affected by the low number of studies.

In the present scenario, therefore, there is some initial evidence supporting the relationship between MPS and measurements of intracortical disinhibition; nevertheless, the present evidence still preliminarily supports a causal relationship between these 2 factors. Indeed, patients with other conditions seem to express the same alterations in cortical excitability than patients with MPS (see section 5), and these markers are influenced by parameters other than pain, such as depression, anxiety, or pain catastrophizing. In addition, the 4 studies evaluating ICI and ICF in patients with MPS did not show the exact same results. The investigator and the clinician should then interpret this evidence as potentially useful to better understand the mechanisms of MPS; however, consideration should be given to the fact that this evidence is still in its early stages.

## 5. Specificity of intracortical disinhibition in myofascial pain syndrome

As discussed above, other chronic pain syndromes such as fibromyalgia^36^ present the same cortical disinhibition pattern as measured by a reduction of ICI and/or an increase in ICF. Fibromyalgia seems to be the closest pathology to MPS and intracortical excitability parameters present the same dysregulation of cortical inhibition in the motor cortex.^8,39^ Interestingly, rTMS-related pain reduction was correlated to modification of ICI,^38^ as shown in patients with MPS.^14^ These 2 musculoskeletal chronic pain syndromes shared pathophysiological similarities that could explain the presence of intracortical disinhibition in both syndromes.^17,18,20^

Although pathophysiological mechanisms of pain are widely different from MPS, modifications in cortical excitability have been identified in patients with limb amputation. Schwenkreis et al (2000) found a significant reduction of ICI coupled with an enhancement of ICF in the affected side: the contralateral hemisphere of the amputated limb.^58^ Note that even if the relationship between pain and cortical disinhibition was not directly investigated, all patients included in that study had an average pain of 3.5 on the visual analog scale.

For chronic pain syndromes with structural lesions such as osteoarthritis, this disinhibition phenomenon was not observed. Indeed, patients with osteoarthritis seem to have the same cortical excitability parameters as healthy controls, as previously described in Caumo et al (2016).^8^ The authors suggested that this difference between patients with MPS or fibromyalgia (scarcity of tissue injury) and patients with osteoarthritis (structural lesion) could be explained by distinct plastic changes in pain pathways. Indeed, in osteoarthritis, pain arises by the activation of primary nociceptive afference from the damaged tissue, whereas in other neuropathic pain syndromes, pain occurs without this activation of nociceptors.^63^ Therefore, a sustained activation of the nociceptive system leads to an involvement of different brain networks as compared to pain occurring in the absence of a clear source of nociception in other chronic pain syndromes with no evident structural lesions.^69^

Another chronic pain syndrome that affects a large proportion of the population is chronic headaches. Few studies have investigated ICI and ICF in patients having chronic migraines. The findings were comparable with those for MPS and fibromyalgia; patients had a reduced CSP^13,28^ or an increase in ICF,^60^ suggesting that patients with chronic headaches have a reduction of inhibitory circuits.

Although most studies on neuropathic pain confirmed these findings, some trials on fibromyalgia and migraine found mixed results. For instance, in fibromyalgia, pain reduction after transcranial direct current stimulation has been found to be linked to a reduction, not an increase, in ICI.^1^ In line with this observation, Mhalla et al (2010) found that patients with fibromyalgia demonstrate both reduced in ICF and ICI as compared to healthy controls.^39^ Similar inconsistencies and opposite results have been found in migraine.^11^ Therefore, we cannot generalize our findings to all chronic pain syndromes for which some inconsistencies have been observed.

Beside chronic pain syndromes' etiology, an important factor to take into account is confounding because patients with chronic pain generally have other symptoms such as depression, anxiety, or fatigue.^7,41,45^ As exposed in 2 studies assessing the effect of pain catastrophizing and anxiety on cortical excitability parameters,^65,66^ these factors were also directly correlated to intracortical disinhibition parameters. In fibromyalgia as well, fatigue, catastrophizing, and depression correlated with higher dysregulation of intracervical inhibition parameters.^38^ Therefore, cortical disinhibition may not be directly related to chronic pain, but the consequence of multiple factors related to this multifaceted condition. Further data may provide the required level of detail to understand potential differences and implications of disinhibition.

## 6. Conclusion

In this systematic review, we identified 4 studies investigating intracortical disinhibition by means of corticospinal excitability (ICI and ICF) in patients with MPS.^3,8,65,66^ Although this is a limited amount of data, all 4 studies reported a decreased ICI and/or an increased ICF, —supporting the intracortical disinhibition theory in this population of patients. In addition, these trials highlighted the association between other common psychological factors in patients with chronic pain,^62^ such as pain catastrophizing or anxiety and intracortical disinhibition measurements, further demonstrating the importance of these neurophysiological factors in chronic pain and related psychological symptoms. The low number of studies we found regarding this matter emphasizes the lack of neurophysiological markers for patients with MPS. However, intracortical disinhibition measurements have been identified in a reproducible way in different population of patients having a chronic pain condition, more specifically in neuropathic pain syndromes.^57^

Although the present data are too preliminary to draw generalizable conclusions, they do support the intracortical disinhibition theory in MPS, although not exclusively for this specific condition. Further prospective trials exploring the relationship between these neurophysiological measurements and the development of chronic pain could shed light on the development of neuroplastic changes in chronic pain syndromes. In addition, larger trials assessing these markers in different populations of patients having chronic pain could help to differentiate and better characterize chronic pain diseases, as well as finding similar aspects. A better understanding of neurophysiological modifications related to chronic pain will give new insights in the development of treatments targeting these maladaptive changes, such as NIBS techniques (eg, rTMS and transcranial direct current stimulation). Finally, intracortical disinhibition measurements could be used as an objective measure of treatment efficacy, as compared to clinical scales, and a potential predictive marker of a response to a treatment.

## Disclosures

The authors have no conflict of interest to declare.

A. Thibaut has been supported by the Wallonie Brussel International scholarship and the Fond Leon Fredericq. F. Fregni has been supported by NIH RO1grant (1R01HD082302-01A1).

## References

[1] Antal A Terney D Kuhnl S Paulus W Anodal transcranial direct current stimulation of the motor cortex ameliorates chronic pain and reduces short intracortical inhibition. J Pain Symptom Manag 2010;39:890–903. 10.1016/j.jpainsymman.2009.09.023 20471549

[2] Avoli M Hwa G Louvel J Kurcewicz I Pumain R Lacaille J Functional and pharmacological properties of GABA-mediated inhibition in the human neocortex. Can J Physiol Pharmacol 1997;75:526–34. 9250388

[3] Botelho LM Morales-Quezada L Rozisky JR Brietzke AP Torres ILS Deitos A Fregni F Caumo W A framework for understanding the relationship between descending pain modulation, motor corticospinal, and neuroplasticity regulation systems in chronic myofascial pain. Front Hum Neurosci 2016;10:1–12. 27445748 10.3389/fnhum.2016.00308 PMC4921500

[4] Camanho L Imamura M Arendt-Nielsen L Genesis of pain in arthrosis. Rev Bras Ortop 2011;46:14–17. 27026978 10.1016/S2255-4971(15)30168-3 PMC4799164

[5] Castillo-Saavedra L Gebodh N Bikson M Diaz-Cruz C Brandao R Coutinho L Truong D Datta A Shani-Hershkovich R Weiss M Laufer I Reches A Peremen Z Geva A Parra LC Fregni F Clinically effective treatment of fibromyalgia pain with high-definition transcranial direct current stimulation: phase II open-label dose optimization. J Pain 2016;17:14–26. 26456677 10.1016/j.jpain.2015.09.009 PMC5777157

[6] Castillo Saavedra L Mendonca M Fregni F Role of the primary motor cortex in the maintenance and treatment of pain in fibromyalgia. Med Hypotheses 2014;83:332–6. 24992875 10.1016/j.mehy.2014.06.007

[7] Castro M Kraychete D Daltro C Lopes J Menezes R Oliveira I Comorbid anxiety and depression disorders in patients with chronic pain. Arq Neuropsiquiatr 2009;67:982–5. 20069205 10.1590/s0004-282x2009000600004

[8] Caumo W Deitos A Carvalho S Leite J Carvalho F Dussán-Sarria JA Tarragó MGL Souza A Torres ILS Fregni F Motor cortex excitability and BDNF levels in chronic musculoskeletal pain according to structural pathology. Front Hum Neurosci 2016;10:357. 27471458 10.3389/fnhum.2016.00357 PMC4946131

[9] Caumo W Ruehlman LS Karoly P Sehn F Vidor LP Dall-Ágnol L Chassot M Torres ILS Cross-cultural adaptation and validation of the profile of chronic pain: screen for a Brazilian population. Pain Med 2013;14:52–61. 23171145 10.1111/j.1526-4637.2012.01528.x

[10] Chen R Corwell B Yaseen Z Hallett M Cohen LG Mechanisms of cortical reorganization in lower-limb amputees. J Neurosci 1998;18:3443–50. 9547251 10.1523/JNEUROSCI.18-09-03443.1998 PMC6792672

[11] Conforto AB Moraes MS Amaro E Young WB Lois LA Gonçalves AL Peres MFP Increased variability of motor cortical excitability to transcranial magnetic stimulation in migraine: a new clue to an old enigma. J Headache Pain 2012;13:29–37. 21881905 10.1007/s10194-011-0379-4 PMC3253159

[12] Cook DB Lange G Ciccone DS Liu WC Steffener J Natelson BH Functional imaging of pain in patients with primary fibromyalgia. J Rheumatol 2004;31:364–78. 14760810

[13] Curra A Pierelli F Coppola G Barbanti P Buzzi MG Galeotti F Serrao M Truini A Casali C Pauri F Cruccu G Shortened cortical silent period in facial muscles of patients with migraine. PAIN 2007;132:124–31. 17574759 10.1016/j.pain.2007.05.009

[14] Dall'Agnol L Medeiros LF Torres ILS Deitos A Brietzke A Laste G de Souza A Vieira JL Fregni F Caumo W Repetitive transcranial magnetic stimulation increases the corticospinal inhibition and the brain-derived neurotrophic factor in chronic myofascial pain syndrome: an explanatory double-blinded, randomized, sham-controlled trial. J Pain 2014;15:845–55. 24865417 10.1016/j.jpain.2014.05.001

[15] Di Lazzaro V Oliviero A Saturno E Dileone M Pilato F Nardone R Ranieri F Musumeci G Fiorilla T Tonali P Effects of lorazepam on short latency afferent inhibition and short latency intracortical inhibition in humans. J Physiol 2005;564:661–8. 15718269 10.1113/jphysiol.2004.061747 PMC1464438

[16] Fedak KM Bernal A Capshaw ZA Gross S Applying the Bradford Hill criteria in the 21st century: how data integration has changed causal inference in molecular epidemiology. Emerg Themes Epidemiol 2015;12:14. 26425136 10.1186/s12982-015-0037-4 PMC4589117

[17] Ge HY Nie H Madeleine P Danneskiold-Samsøe B Graven-Nielsen T Arendt-Nielsen L Contribution of the local and referred pain from active myofascial trigger points in fibromyalgia syndrome. PAIN 2009;147:233–40. 19819074 10.1016/j.pain.2009.09.019

[18] Ge HY Wang Y Danneskiold-Samsøe B Graven-Nielsen T Arendt-Nielsen L The predetermined sites of examination for tender points in Fibromyalgia Syndrome are frequently associated with myofascial trigger points. J Pain 2010;11:644–51. 19914876 10.1016/j.jpain.2009.10.006

[19] Gerwin RD Classification, epidemiology, and natural history of myofascial pain syndrome. Curr Pain Headache Rep 2001;5:412–20. 11560806 10.1007/s11916-001-0052-8

[20] Gerwin RD A review of myofascial pain and fibromyalgia—factors that promote their persistence. Acupunct Med 2005;23:121–34. 16259310 10.1136/aim.23.3.121

[21] Gerwin RD Differential diagnosis of myofascial pain syndrome and fibromyalgia. J Musculoskelet Pain 1999;7:209–15.

[22] Harden R Muscle Pain Syndromes. Am J Phys Med Rehabil 2007;86:S47–58. 17370371 10.1097/phm.0b013e31802ba648

[23] Henderson LA Peck CC Petersen ET Rae CD Youssef AM Reeves JM Wilcox SL Akhter R Murray GM Gustin SM Chronic Pain: lost Inhibition? J Neurosci 2013;33:7574–82. 23616562 10.1523/JNEUROSCI.0174-13.2013 PMC6619566

[24] Hill B The environment and disease: association or causation? Proc R Soc Med 1965;58:295–300. 14283879 10.1177/003591576505800503 PMC1898525

[25] Inghillerj M Berardelli A Cruccu G Manfredi M Silent period evoked by transcranial stimulation of the human cortex and cervicomedullary junction. J Physiol 1993;466:521–34. 8410704 PMC1175490

[26] Jafri MS Mechanisms of myofascial pain. Int Sch Res Notices 2014;2014:1–16. 10.1155/2014/523924 PMC4285362 25574501

[27] Kapogiannis D Wassermann EM Transcranial magnetic stimulation in Clinical Pharmacology. Cent Nerv Syst Agents Med Chem 2008;8:234–40. 19122782 10.2174/187152408786848076 PMC2613312

[28] Khedr EM Ahmed MA Mohamed KA Motor and visual cortical excitability in migraineurs patients with or without aura: transcranial magnetic stimulation. Neurophysiol Clin 2006;36:13–18. 16530139 10.1016/j.neucli.2006.01.007

[29] Kramer PR Bellinger LL Reduced GABAA receptor alpha6 expression in the trigeminal ganglion enhanced myofascial nociceptive response. Neuroscience 2013;245:1–11. 23602886 10.1016/j.neuroscience.2013.04.003 PMC3740526

[30] Latremoliere A Woolf CJ Central sensitization: a generator of pain hypersensitivity by Central Neural Plasticity. J Pain 2009;10:895–926. 19712899 10.1016/j.jpain.2009.06.012 PMC2750819

[31] Lautenbacher S Rollman GB Possible deficiencies of pain modulation in fibromyalgia. Clin J Pain 1997;13:189–96. 9303250 10.1097/00002508-199709000-00003

[32] Lefaucheur J Cortical neurostimulation for neuropathic pain: state of the art and perspectives. PAIN 2016;157(suppl 1):S81–9. 26785160 10.1097/j.pain.0000000000000401

[33] Lefaucheur JP Antal A Ahdab R Ciampi de Andrade D Fregni F Khedr EM Nitsche M Paulus W The use of repetitive transcranial magnetic stimulation (rTMS) and transcranial direct current stimulation (tDCS) to relieve pain. Brain Stimul 2008;1:337–44. 20633392 10.1016/j.brs.2008.07.003

[34] Lefaucheur JP Drouot X Ménard-Lefaucheur I Keravel Y Nguyen JP Motor cortex rTMS restores defective intracortical inhibition in chronic neuropathic pain. Neurology 2006;67:1568–74. 17101886 10.1212/01.wnl.0000242731.10074.3c

[35] Lenz M Höffken O Stude P Lissek S Schwenkreis P Reinersmann A Frettlöh J Richter H Tegenthoff M Maier C Bilateral somatosensory cortex disinhibition in complex regional pain syndrome type I. Neurology 2011;77:1096–101. 21880999 10.1212/WNL.0b013e31822e1436

[36] Lim M Roosink M Kim JS Kim DJ Kim HW Lee EB Kim HA Chung CK Disinhibition of the primary somatosensory cortex in patients with fibromyalgia. PAIN 2015;156:666–74. 25630027 10.1097/j.pain.0000000000000096

[37] Matsunaga K Akamatsu N Uozumi T Urasaki E Tsuji S Early and late inhibition in the human motor cortex studied by paired stimulation through subdural electrodes. Clin Neurophysiol 2002;113:1099–109. 12088706 10.1016/s1388-2457(02)00079-2

[38] Mhalla A Baudic S De Andrade DC Gautron M Perrot S Teixeira MJ Attal N Bouhassira D Long-term maintenance of the analgesic effects of transcranial magnetic stimulation in fibromyalgia. PAIN 2011;152:1478–85. 21397400 10.1016/j.pain.2011.01.034

[39] Mhalla A de Andrade DC Baudic S Perrot S Bouhassira D Alteration of cortical excitability in patients with fibromyalgia. PAIN 2010;149:495–500. 20356675 10.1016/j.pain.2010.03.009

[40] Michels L Christidi F Steiger VR Sandor PS Gantenbein AR Landmann G Schreglmann SR Kollias S Riederer F Pain modulation is affected differently in medication-overuse headache and chronic myofascial pain—A multimodal MRI study. Cephalalgia 2016;0:1–16. 10.1177/0333102416652625 27250235

[41] Morin CM Gibson D Wade J Self-reported sleep and mood disturbance in chronic pain patients. Clin J Pain 1998;14:311–14. 9874009 10.1097/00002508-199812000-00007

[42] Morton DL Sandhu JS Jones AKP Brain imaging of pain: state of the art. J Pain Res 2016;9:613–24. 27660488 10.2147/JPR.S60433 PMC5019436

[43] Nakamura H Kitagawa H Kawaguchi Y Tsuji H Intracortical facilitation and inhibition after transcranial magnetic stimulation in conscious humans. J Physiol 1997;498(pt 3):817–23. 9051592 10.1113/jphysiol.1997.sp021905 PMC1159197

[44] Neblett R Cohen H Choi Y Hartzell MM Williams M Mayer TG Gatchel RJ The central sensitization inventory (CSI): establishing clinically significant values for identifying central sensitivity syndromes in an outpatient chronic pain sample. J Pain 2013;14:438–45. 23490634 10.1016/j.jpain.2012.11.012 PMC3644381

[45] Nicholson B Verma S Comorbidities in chronic neuropathic pain. Pain Med 2004;5:(suppl 1):S9–S27. 14996227 10.1111/j.1526-4637.2004.04019.x

[46] Nitsche MA Monte-Silva K Kuo MF Paulus W Dopaminergic impact on cortical excitability in humans. Rev Neurosci 2010;21:289–98. 21086761 10.1515/revneuro.2010.21.4.289

[47] Petersen NT Pyndt HS Nielsen JB Investigating human motor control by transcranial magnetic stimulation. Exp Brain Res 2003;152:1–16. 12879177 10.1007/s00221-003-1537-y

[48] Peyron R Laurent B Garcia-Larrea L Functional imaging of brain responses to pain. A review and meta-analysis (2000). Neurophysiol Clin 2000;30:263–88. 11126640 10.1016/s0987-7053(00)00227-6

[49] Pinheiro ESDS de Queiros FC Montoya P Santos CL do Nascimento MA Ito CH Silva M Nunes Santos DB Benevides S Miranda JGV Sa KN Baptista AF Electroencephalographic patterns in chronic pain: a systematic review of the literature. PLoS One 2016;11:e0149085. 26914356 10.1371/journal.pone.0149085 PMC4767709

[50] Pujol J López-Solà M Ortiz H Vilanova JC Harrison BJ Yücel M Soriano-Mas C Cardoner N Deus J Mapping brain response to pain in fibromyalgia patients using temporal analysis of fMRI. PLoS One 2009;4:e5224. 19381292 10.1371/journal.pone.0005224 PMC2667672

[51] Radhu N de Jesus DR Ravindran LN Zanjani A Fitzgerald PB Daskalakis ZJ A meta-analysis of cortical inhibition and excitability using transcranial magnetic stimulation in psychiatric disorders. Clin Neurophysiol 2013;124:1309–20. 23485366 10.1016/j.clinph.2013.01.014

[52] Rossini PM Barker AT Berardelli A Caramia MD Caruso G Cracco RQ DimitrijeviImage MR Hallett M Katayama Y Lücking CH Maertens de Noordhout AL Marsden CD Murray NMF Rothwell JC Swash M Tomberg C Non-invasive electrical and magnetic stimulation of the brain, spinal cord and roots: basic principles and procedures for routine clinical application. report of an IFCN Committee. Electroencephalogr Clin Neurophysiol 1994;91:2198–208. 10.1016/0013-4694(94)90029-9 7519144

[53] Ruehlman LS Karoly P Newton C Aiken LS The development and preliminary validation of a brief measure of chronic pain impact for use in the general population. PAIN 2005;113:82–90. 15621367 10.1016/j.pain.2004.09.037

[54] Salerno A Thomas E Olive P Blotman F Picot MC Georgesco M Motor cortical dysfunction disclosed by single and double magnetic stimulation in patients with fibromyalgia. Clin Neurophysiol 2000;111:994–1001. 10825705 10.1016/s1388-2457(00)00267-4

[55] Sandrini G Rossi P Milanov I Serrao M Cecchini AP Nappi G Abnormal modulatory influence of diffuse noxious inhibitory controls in migraine and chronic tension-type headache patients. Cephalalgia 2006;26:782–9. 16776692 10.1111/j.1468-2982.2006.01130.x

[56] Schwenkreis P Janssen F Rommel O Pleger B Völker B Hosbach I Dertwinkel R Maier C Tegenthoff M Bilateral motor cortex disinhibition in complex regional pain syndrome (CRPS) type I of the hand. Neurology 2003;61:515–19. 12939426 10.1212/wnl.61.4.515

[57] Schwenkreis P Scherens A Ronnau AK Hoffken O Tegenthoff M Maier C Cortical disinhibition occurs in chronic neuropathic, but not in chronic nociceptive pain. BMC Neurosci 2010;11:73. 20540759 10.1186/1471-2202-11-73 PMC2898830

[58] Schwenkreis P Witscher K Janssen F Dertwinkel R Zenz M Malin JP Tegenthoff M Changes of cortical excitability in patients with upper limb amputation. Neurosci Lett 2000;293:143–6. 11027854 10.1016/s0304-3940(00)01517-2

[59] Siebner HR Dressnand J Auer C Conrad B Continuous intrathecal baclofen infusions induced a marked increase of the transcranially evoked silent period in a patient with generalized dystonia. Muscle Nerve 1998;21:1209–12. 9703450 10.1002/(sici)1097-4598(199809)21:9<1209::aid-mus15>3.0.co;2-m

[60] Siniatchkin M Kröner-Herwig B Kocabiyik E Rothenberger A Intracortical inhibition and facilitation in migraine–a transcranial magnetic stimulation study. Headache 2007;47:364–70. 17371353 10.1111/j.1526-4610.2007.00727.x

[61] Stetkarova I Kofler M Differential effect of baclofen on cortical and spinal inhibitory circuits. Clin Neurophysiol 2013;124:339–45. 22877625 10.1016/j.clinph.2012.07.005

[62] Sullivan MJL Rodgers WM Kirsch I Catastrophizing, depression and expectancies for pain and emotional distress. PAIN 2001;91:147–54. 11240087 10.1016/s0304-3959(00)00430-9

[63] Treede RD Jensen TS Campbell JN Cruccu G Dostrovsky JO Griffin JW Hansson P Hughes R Nurmikko T Serra J Neuropathic pain: redefinition and a grading system for clinical and research purposes. Neurology 2008;70:1630–5. 18003941 10.1212/01.wnl.0000282763.29778.59

[64] Treede RD Kenshalo DR Gracely RH Jones AKP The cortical representation of pain. PAIN 1999;79:105–11. 10068155 10.1016/s0304-3959(98)00184-5

[65] Vidor LP Torres ILS Medeiros LF Dussán-Sarria JA Dall'agnol L Deitos A Brietzke A Laste G Rozisky JR Fregni F Caumo W Association of anxiety with intracortical inhibition and descending pain modulation in chronic myofascial pain syndrome. BMC Neurosci 2014;15:42. 24645677 10.1186/1471-2202-15-42 PMC3995110

[66] Volz MS Medeiros LF Tarragô MDG Vidor LP Dall'Agnol L Deitos A Brietzke A Rozisky JR Rispolli B Torres ILS Fregni F Caumo W The relationship between cortical excitability and pain catastrophizing in myofascial pain. J Pain 2013;14:1140–7. 23810270 10.1016/j.jpain.2013.04.013

[67] Volz MS Suarez-Contreras V Portilla ALS Illigens B Bermpohl F Fregni F Movement observation-induced modulation of pain perception and motor cortex excitability. Clin Neurophysiol 2015;126:1204–11. 25308309 10.1016/j.clinph.2014.09.022

[68] Woolf CJ Central sensitization: implications for the diagnosis and treatment of pain. PAIN 2012;152:1–31. 10.1016/j.pain.2010.09.030 PMC3268359 20961685

[69] Woolf CJ Salter MW Neuronal plasticity: increasing the gain in pain. Science 2000;288:1765–9. 10846153 10.1126/science.288.5472.1765

[70] Ziemann U Lonnecker S Steinhoff BJ Paulus W Effects of antiepileptic drugs on motor cortex excitability in humans—a Transcranial Magnetic Stimulation Study. Ann Neurol 1996;40:367–78. 8797526 10.1002/ana.410400306

[71] Ziemann U Lönnecker S Steinhoff BJ Paulus W Ziemann U Steinhoff W Paulus BJ The effect of Iorazepam on the motor cortical excitability in man. Exp Brain Res 1996;109:127–35. 8740215 10.1007/BF00228633

[72] Ziemann U Rothwell JC Ridding MC Interaction between intracortical inhibition and facilitation in human motor cortex. J Physiol 1996;496(pt 3):873–81. 8930851 10.1113/jphysiol.1996.sp021734 PMC1160871

